# Downregulation of DAB2IP Promotes Mesenchymal-To-Neuroepithelial Transition and Neuronal Differentiation of Human Mesenchymal Stem Cells

**DOI:** 10.1371/journal.pone.0075884

**Published:** 2013-09-20

**Authors:** Sunny Li-Yun Chang, Ruey-Hwang Chou, Hong-Jie Zeng, Yu-Hsuan Lin, Tai-Yu Chiu, De-Ming Yang, Shih-Chieh Hung, Chih-Ho Lai, Jer-Tsong Hsieh, Woei-Cherng Shyu, Yung-Luen Yu

**Affiliations:** 1 Graduate Institute of Basic Medical Science, and Graduate Institute of Molecular Systems Biomedicine, China Medical University, Taichung, Taiwan; 2 Graduate Institute of Cancer Biology, and Center for Molecular Medicine, China Medical University, Taichung, Taiwan; 3 Department of Biotechnology, Asia University, Taichung, Taiwan; 4 Department of Medical Research and Education, Taipei Veterans General Hospital, Taipei, Taiwan; 5 Institute of Biophotonics, School of Medical Technology and Engineering and Biophotonics and Molecular Imaging Research Center, National Yang-Ming University, Taipei, Taiwan; 6 Stem Cell Laboratory, Department of Medical Research and Education, Orthopaedics and Traumatology, Taipei Veterans General Hospital and Institute of Clinical Medicine, Institute of Pharmacology, National Yang-Ming University, Taipei, Taiwan; 7 Department of Microbiology, School of Medicine, Graduate Institute of Basic Medical Science, China Medical University, Taichung, Taiwan; 8 University of Texas, Department of Urology, Southwestern Medical Center, Dallas, Texas, United States of America; 9 Graduate Institute of Immunology, China Medical University, Taichung, Taiwan; 10 Translational Medicine Research Center and Center for Neuropsychiatry, China Medical University Hospital, Taichung, Taiwan; Rutgers - New Jersey Medical School, United States of America

## Abstract

The DOC-2/DAB2 interactive protein (DAB2IP) is a new member of the Ras GTPase–activating protein family. Recent studies have shown that, in addition to its tumor suppressive role in various tumors, DAB2IP also plays an important role in regulating neuronal migration and positioning during brain development. In this study, we determined the roles of DAB2IP in the neuronal differentiation of human mesenchymal stem cells (hMSCs). We found that lentiviral short hairpin RNA (shRNA)-mediated knockdown of DAB2IP promoted the mesenchymal-to-neuroepithelial stem cell transition (MtNeST) and neuronal differentiation, which were accompanied by a reduction of cell proliferation but not apoptosis or cellular senescence. This suggests that DAB2IP plays an important role in the neuronal induction of hMSCs. Moreover, our finding that reduction of glycogen synthase kinase 3 beta (GSK3β) activity upon LiCl pretreatment inhibited both the MtNeST and production of MAP2-positive cells upon DAB2IP knockdown suggests that this transition is most likely mediated by regulation of the GSK3β signaling pathway. Our study demonstrates that DAB2IP participates in the first step of neuron induction of hMSCs, which implies a potentially important role for DAB2IP in the MtNeST during neurogenesis.

## Introduction

Deletion of ovarian carcinoma 2/disabled homolog 2 (DOC-2/DAB2) interactive protein (DAB2IP), also known as apoptosis signal-regulating kinase–interacting protein-1, is a new member of the Ras GTPase-activating protein family [1]. Previous studies of DAB2IP have unveiled its tumor suppressor role in various types of cancers [1-5]. DAB2IP can inhibit cell survival by suppressing the phosphatidyl inositol 3-kinase (PI3K)-Akt kinase signaling pathway and can also promote tumor necrosis factors mediated apoptosis by activating the apoptosis signal–regulating kinase (ASK1)-c-Jun N-terminal kinase (JNK) pathway [6,7]. In general, loss of DAB2IP expression rvia epigenetic silencing during tumorigenesis is associated with the poor prognosis and tumor metastasis [2-4,8,9].

In addition to its tumor suppressor role, DAB2IP also plays an important role in regulating brain development. Mouse *Dab2ip* is widely expressed in embryonic and adult brains, and high expression of *Dab2ip* has been observed in the cerebral cortex, hippocampus, midbrain, and cerebellum [10]. *Dab2ip* function in the developing mouse brain is likely related to its interacting proteins, *Drosophila* disabled homolog 1 and 2 (Dab1 and Dab2). *Dab1* has restricted expression in the developing central nervous system [11] and is an important cytoplasmic adaptor protein of Reelin signaling [12]. Targeted inactivation and spontaneous mutations of *Dab1* in mice generate a *reeler*-like phenotype [12-14]. Many reports have established a role for Reelin-Dab1 signaling in regulating neuronal migration and positioning during brain development [8,15,16]. *Dab2*, although not as restricted as *Dab1*, is also expressed in embryonic brains [17]. Studies in rat pheochromocytoma PC12 cells have demonstrated that Dab2 negatively regulates of nerve growth factor–mediated neurite outgrowth [17]. In addition, recent studies have shown that mouse *Dab2ip* plays crucial roles in neurite growth and stabilization and in neuronal migration in the developing neocortex [18]. *Dab2ip* also regulates dendritic development and synapse formation in the developing cerebellum [19]. Although the neurophysiological functions of *Dab2ip* in cortical and cerebellar development have been revealed, the underlying molecular mechanisms remain unclear.

Human mesenchymal stem cells (hMSCs) are multipotent and capable of differentiating into different cell types, including neurons, which cannot be regenerated [20-23]. Over the past several years, strategies for hMSC-based cellular therapy have been applied in animal models and human subjects with neurodegenerative diseases, such as Alzheimer’s and Parkinson’s diseases [24-26]. In this study, we demonstrate a new role for *DAB2IP* in the neuronal differentiation of bone marrow–derived hMSCs. Our results implicate multiple functional roles of *DAB2IP* in cell cycle arrest, neuronal differentiation of the hMSCs and the mesenchymal-to-neuroepithelial stem cell transition (MtNeST), in which glycogen synthase kinase- 3β (GSK3β) signaling is involved.

## Materials and Methods

### Ethics statement

The study protocol was approved by the Institutional Animal Care and Use Committee of China Medical University and Hospital (No. 99-37-N).

### Culture of hMSCs and induction of differentiation

Primary hMSCs, size-sieved from human bone marrow, were previously isolated and characterized as having multilineage potential to form bone, fat, cartilage [27], and electrically active neural cells [28]. The human papillomavirus 16 E6/E7-immortalized derivative 3A6-hMSCs, which contain the human telomerase reverse transcriptase gene for more stem-like properties [29], were used in this study. hMSCs were maintained in growth medium, low-glucose Dulbecco’s Modified Eagle’s Medium (LG-DMEM, HyClone) supplemented with 10% fetal bovine serum, 100 U/ml penicillin and 100 µg/ml streptomycin, in a 37°C humidified incubator with 5% CO_2_. Neuronal induction procedures were performed as described [22,30]. After culture for 24 h, hMSCs were seeded at a density of 4000 cells/cm^2^ in 100-mm cell culture dishes or 24-well cell culture plates and incubated for 1 to 5 days with neuronal induction medium (NIM) containing 3-isobutyl-1-methyl-xanthine, a non-specific phosphodiesterase inhibitor [22,30]. The NIM was changed on the third day. For experiments with LiCl, MSCs were pretreated with or without 40 mM LiCl in NIM for 16 h, and then cultured in NIM for 24 h.

### DAB2IP knockdown via lentivirus carrying a DAB2IP-specific short hairpin RNA (shRNA)

The shRNA target sequences were as follows: negative control luciferase shRNA (shLuc, TRCN0000072244): 5’-ATCACAGAATCGTCGTATGCA-3’; DAB2IP shRNA #57 (sh57, TRCN0000001457): 5’-GTAATGTAACTATCTCACCTA-3’ (in the coding region), shRNA #59 (sh59, TRCN0000001459): 5’-GAGTTCATCAAAGCGCTGTAT-3’ (in the 3’-untranslated region). Knockdown (KD) of DAB2IP expression by lentivirus-mediated shRNA in 3A6-hMSCs was performed according to instructions from the National RNAi Core Facility (Academia Sinica, Taipei, Taiwan) [31,32].

To prepare lentivirus transduction particles, HEK293T cells in 100-mm cell culture dishes were co-transfected with 2 µg pCMV-R8.91 harboring Gag and Pol genes, 0.2 µg pMD.G containing the gene for expressing the vesicular stomatitis virus envelope glycoprotein (i.e., VSV-G), and 2 µg pLKO.1 bearing specific shRNAs. Cells were incubated in transfection medium (OPTI/MEM, Gibco) for 6 h, followed by incubation in DMEM/F12 (HyClone) supplemented with 10% fetal bovine serum, 100 U/ml penicillin, 100 µg/ml streptomycin, and 1% bovine serum albumin for 24 h. The culture medium containing lentivirus particles was collected, aliquoted, and stored at -80°C.

For lentivirus transduction, hMSCs were cultured until they reached approximately 80% confluency, after which they were infected with lentivirus-bearing specific shRNAs in growth medium containing 8 µg/ml polybrene for 24 h. Infected hMSCs were subcultured for 3 days in growth medium for immunofluorescence staining of β-tubulin III (Tuj-1), semi-quantitative reverse transcription (RT)-PCR analysis of neuron-specific enolase (NSE), and western blotting for GSK3β. For measurement of microtubule-associated protein 2 (MAP2)-positive cells by flow cytometry, infected hMSCs were subcultured in growth medium for 5 days. All subculture media contained 2 µg/ml puromycin for selection of lentivirus-infected cells.

### Semi-quantitative RT-PCR

Total RNA from hMSCs was prepared using ***TRIzol***® ***Reagent*** (Invitrogen). The complementary DNA (cDNA) was synthesized from 2 µg total RNA using oligo dT primer and MML-V reverse transcriptase (Invitrogen); 2 µl cDNA was used as template for each PCR reaction. The specific primers used were as follows: *NSE*: 5’-CATCGACAAGGCTGGCTACACG-3’ (forward), 5’-GACAGTTGCAGGCCTTTTCTTC-3’ (reverse); *DAB2IP*: 5’-TGGACGATGTGCTCTATGCC-3’ (forward), 5’-GGATGGTGATGGTTTGGTAG-3’ (reverse); *Osteopontin* (*OPN*): 5’-ACGCCGACCAAGGAAAACTC-3’ (forward), 5’-GTCCATAAACCACACTATCACCTCG-3’ (reverse); and *β-actin*: 5’-GCACTCTTCCAGCCTTCCTTCC-3’ (forward), 5’-TCACCTTCACCGTTCCAGTTTTT-3’ (reverse). The number of amplification cycles for NSE, DAB2IP, and β-actin was 30, 30, and 25, respectively and were performed as described [33,34].

### Immunoprecipitation, western blotting, and immunofluorescence microscopy

Immunoprecipitation, western blotting, and immunofluorescence analyses were performed as described [22,35,36]. Antibodies against the following proteins were used: β-actin (1:5000, Sigma), CD105 (1:2000, BD Biosciences), DAB2IP (1:2000 for western blotting and 1:1000 for immunofluorescence, Abcam), GSK3β (1:2000, Cell Signaling), pGSK3β (1:2000, Cell Signaling), Ki67 (1:1000, Novocastra), MAP2 (1:2000, BD Biosciences), Nestin (1:2000, Millipore), p16^Ink4a^ (1:2000, Cell Signaling), p21^Cip1^ (1:2000, Cell Signaling) and β-tubulin III (Tuj-1,1:2000, Sigma). For immunofluorescence, cultured 3A6-hMSCs were fixed in 4% paraformaldehyde in phosphate-buffered saline for 15 min. A biotinylated secondary antibody (Vector Labs), elite ABC kit (Vector Labs), and TSA-FITC kit (PerkinElmer) were used for signal detection. Nuclear staining with DAPI (4',6-diamidino-2-phenylindole) was performed after all other staining. Immunofluorescence was analyzed using an Axioplan Fluorescence Trinocular ***Microscope*** (Carl Zeiss MicroImaging).

### Cell proliferation assay and flow cytometric analysis

After culture in puromycin-containing growth medium for 2 days, lentivirus-infected hMSCs were seeded in 96-well cell culture plates (5000 cells/well) for the WST-1 cell proliferation assay (ScienCell). Briefly, on the third day of culture, cells were incubated with WST-1 reagent at 37°C for 4 h, and absorbance (450 nm/630 nm) was measured using a microplate reader (Synergy2, BioTek) as described [37,38]. For MAP2 flow cytometry analysis, hMSCs subcultured for 5 days were trypsinized, fixed, and incubated with the Alexa Fluor® 488-conjugated antibody against MAP2 (BD Biosciences) on ice for 30 min. FACSCalibur flow cytometry with CellQuest™ software (BD Biosciences) was used for analyzing the percentage of MAP2-positive cells.

### Apoptosis assay

To assess apoptosis using annexin V staining, cells were treated with 6 µM camptothecin for 4 h to induce apoptosis, harvested, resuspended and then incubated with FITC-conjugated annexin V and propidium iodide, according to the manufacturer’s protocol (BD Biosciences) and was performed as described [36]. The percentage of apoptotic cells was assessed by flow cytometry.

### Neuronal differentiation from hMSCs in animal brains

Before implantation, hMSCs with or without DAB2IP KD were incubated with 1 µg/ml bisbenzimide Hoechst 33342 (Sigma-Aldrich) for 5 h at 37°C to label nuclei with blue fluorescence as described [22,23].

### Ca^2+^ influx ([Ca^2+^]i) measurement

Ca^2+^ influx was measured as described [22,28]. After treatment, cells were incubated with 5 µM Fura-2 AM for 1 h and then excess Fura-2 AM was washed out. The excitation peak for Fura-2 shifts from 380 nm for the calcium-free chelator to ~340 nm for the calcium-saturated form.

## Results

### Induction of neuronal differentiation in hMSCs

**Figure 1:**
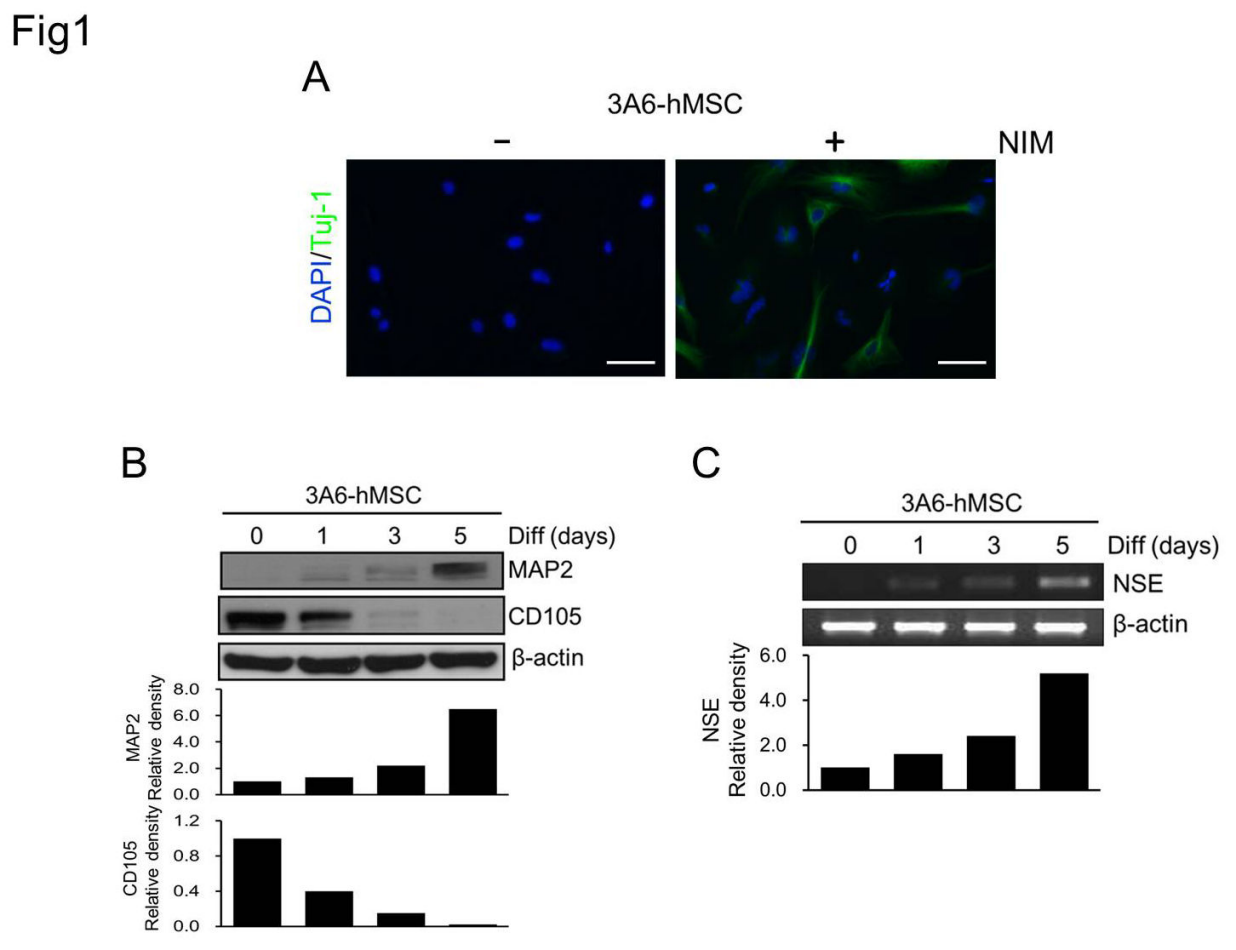
Cell fate transformation and neuronal induction of 3A6-hMSCs by NIM. A. Detection of neuron-specific Tuj-1 expression in differentiating 3A6-hMSCs cultured in NIM for 5 days. Note that Tuj-1 is expressed in the soma and also in induced neurites. Scale bars, 50 µm. B, C. Induced expression of neuron-specific MAP2 (B) and NSE (C) and reduced expression of the mesenchymal cell marker, CD105 (B), during 3A6-hMSC neuronal differentiation (Diff). Western blot and semi-quantitative RT-PCR analyses, respectively. β-actin expression served as an internal control. The bar graphs (bottom) represent the relative density of the MSC marker (CD105), neuron marker (MAP2), and NSE determined by scanning densitometric tracings.

In an attempt to develop *in vitro* culture systems to generate neuronal lineages for the treatment of neurodegenerative diseases, several NIM or culture protocols for specific stem cells have recently been developed [22,29]. Our laboratory and others have reported that hMSCs can be induced to differentiate into functional neuronal cell with electrophysiological properties using NIM containing 3-isobutyl-1-methyl-xanthine [22,23,30]. To further confirm that hMSCs have the potential to differentiate into neuronal cells, different approaches and neuron markers were used to validate the hMSC-derived neuronal cells. Expression of the neuronal markers Tuj-1, MAP2, and NSE was induced in neurons differentiated from 3A6-hMSCs (3A6-hMSC-differentiated neurons) (Figure 1A-C). MAP2 and NSE expression was initiated on the first day of induction and gradually increased during the induction period (Figure 1B and C). In contrast, expression of the MSC marker, CD105, gradually decreased and disappeared on the fifth day of induction (Figure 1B). Notably, induced Tuj-1 expression was detected in the soma and neurites of 3A6-hMSC-differentiated neurons (Figure 1A). These results implicate that the NIM-induced neuronal differentiation of 3A6-hMSCs promoted normal differentiation of neurons, as illustrated by the observed expression pattern of neuronal proteins.

### DAB2IP expression decreases during the neuronal induction of hMSCs

**Figure 2:**
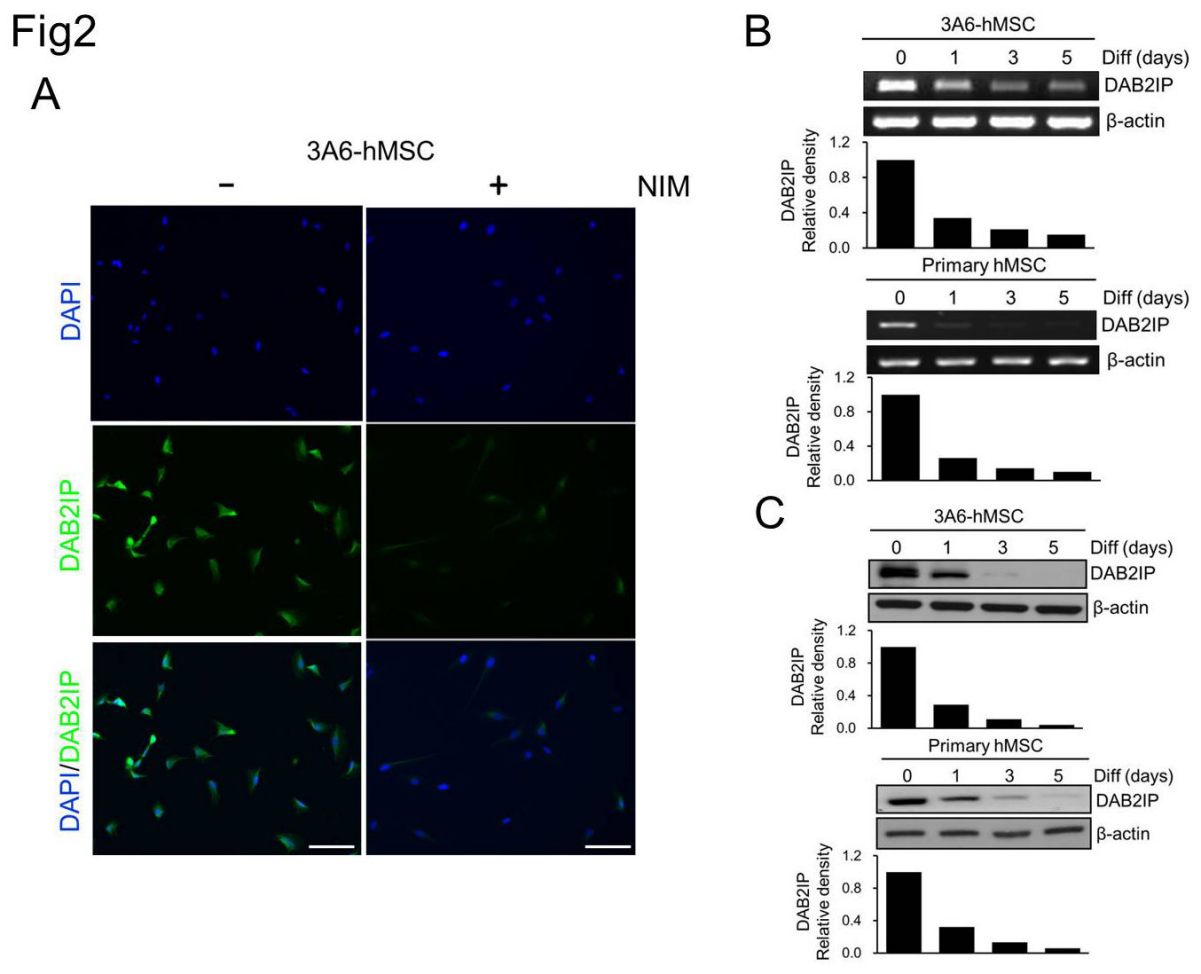
Reduction of DAB2IP expression during the neuronal induction of hMSCs. A. DAB2IP protein expression was readily detected in most of the 3A6-hMSCs (left), whereas in the NIM cells, DAB2IP expression was significantly reduced (right). Images were acquired under the same exposure conditions. B and C. *DAB2IP* expression gradually decreased during the neuronal induction of 3A6-hMSCs and primary hMSCs at both the mRNA (B, RT-PCR) and protein (C, western blot) levels. β-actin served as an internal control. The bar graphs (bottom) represent the relative density of DAB2IP as determined by scanning densitometric tracings.

To investigate the possible involvement of DAB2IP in the neuronal differentiation of hMSCs, we first analyzed DAB2IP expression during neuronal induction of 3A6-hMSCs and primary hMSCs (Figure 2). In naïve 3A6-hMSCs, a basal level of DAB2IP was mainly detected in cytosol (Figure 2A, left panel). One day after NIM, the expression of DAB2IP decreased dramatically either at the mRNA or protein level (Figure 2A right panel, B, and C). These results showed that DAB2IP is present in proliferating hMSCs, and its decreased expression is associated with hMSC differentiation into neurons.

### Downregulation of DAB2IP in hMSCs promotes neuronal differentiation

**Figure 3:**
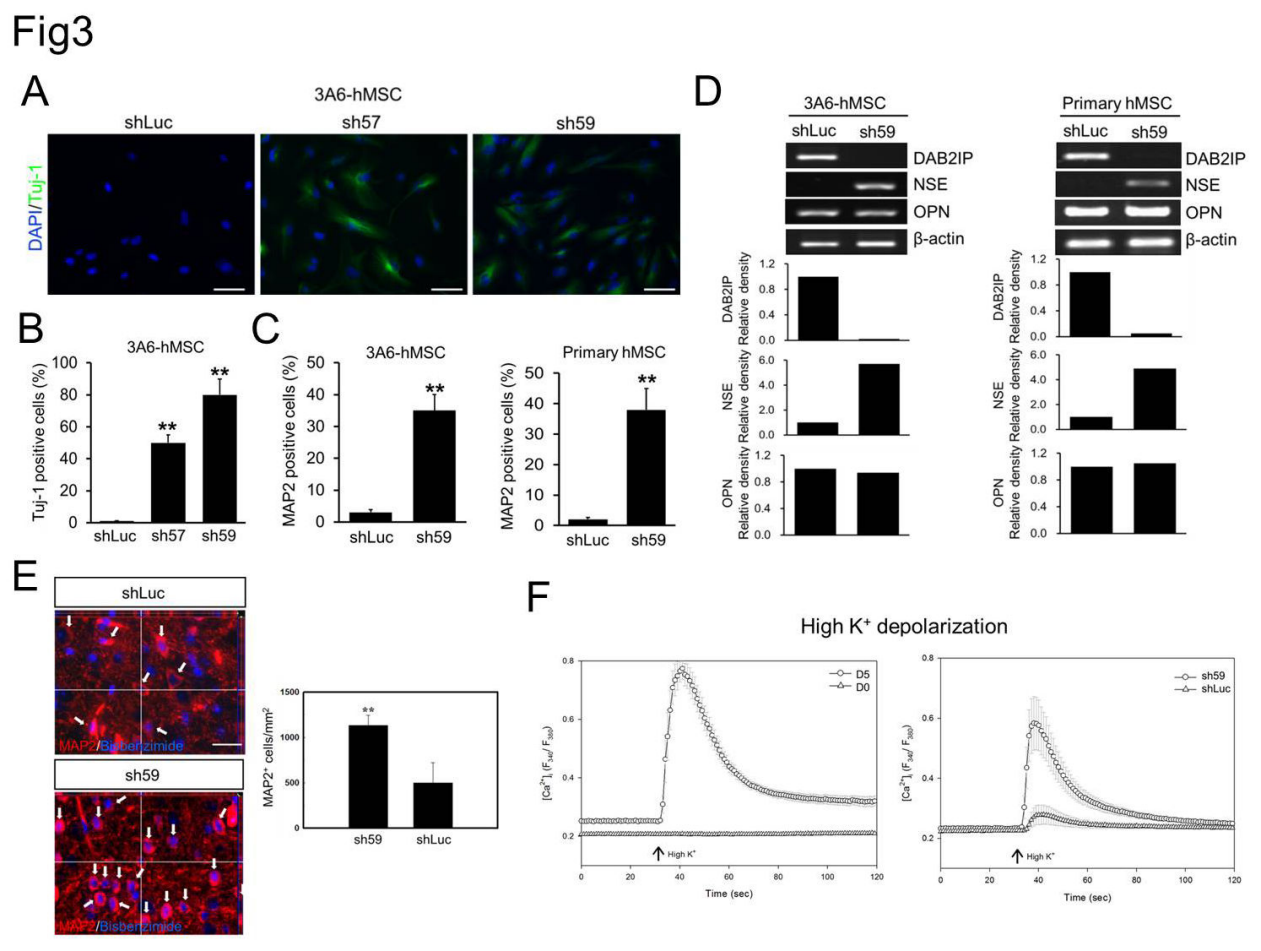
Effect of DAB2IP expression on neuronal differentiation of hMSCs. A. Immunofluorescence staining of Tuj-1 expression (green) in 3A6-hMSCs infected with lentivirus-containing shRNAs against luciferase (shLuc) or DAB2IP (sh57 and sh59). B. Quantification of data presented in A as a percentage of Tuj-1-positive cells relative to the total number of cells. Bars represent mean ± SEM (**p < 0.01, *t* test). C. Flow cytometry of MAP2-positive DAB2IP-KD hMSCs. Bars represent mean ± SEM (**p < 0.01, *t* test). D. Semi-quantitative RT-PCR analysis of DAB2IP, OPN and NSE expression in shRNA-expressing hMSCs. β-actin served as an internal control. The bar graphs (bottom) represent the relative density of DAB2IP, NSE, and OPN as determined by scanning densitometric tracings. E. A representative three-dimensional image of bisbenzimide-labeled primary hMSCs (shLuc and sh59; blue fluorescence) implanted in rat brain. The white arrows indicate the implanted MAP2-positive hMSCs (red fluorescence) in rat brain (left). Scale bar: 50 µm. Quantification of implanted MAP2-positive cells in both the DAB2IP-KD sh59-treated rats and control mock shLuc-treated rats (right). Bars represent mean ± SEM (**p < 0.01, *t* test). F. The primary hMSCs were treated with NIM (D5; circle) or without NIM (D0; triangle) for 5 days (left) and with shLuc or DAB2IP-KD sh59 (right) to induce differentiation and to the neuronal lineage. A change in [Ca^2+^]i after stimulation with high K^+^ buffer was used to verify neuron-like function. The arrow indicates the time point of stimulation.

We then investigated the impact of downregulated DAB2IP expression on the neuronal differentiation of hMSCs. Use of the lentivirus-mediated shRNA gene KD system revealed that decreased DAB2IP expression in 3A6-hMSCs was associated with the appearance of a neuronal-like differentiation phenotype (Figure 3A). Furthermore, the 3A6-hMSCs were subcultured for 3 days in growth medium containing puromycin for selecting stable DAB2IP-KD cells. Based on morphology, the newly generated 3A6-hMSC DAB2IP-KD cells had not completely undergone the transformation to neurites of functional neurons; however, strong Tuj-1-immunoreactive signals were detected in these DAB2IP-KD cells (Figure 3A). Quantification of immunoreactivity showed that Tuj-1-positive cells accounted for ~49% (sh57) and ~78% (sh59) of the shRNA-treated 3A6-hMSCs. This was significantly higher than in control cells expressing shLuc, in which only 2% of cells were positive for Tuj-1 (Figure 3B).

In addition to Tuj-1 expression, MAP2 and NSE expression was also induced in DAB2IP-KD 3A6-hMSCs and primary hMSCs, but genes from other lineages examined were not expressed, e.g., osteopontin (OPN; osteogenic marker; Figure 3C and D). These results suggest that knocking down DAB2IP promotes the differentiation of hMSCs preferentially to the neuronal lineage without NIM. In addition, the mRNA of DAB2IP expression did not significantly change on both hMSCs-differentiated osteogenic lineages [39] or neuroblastoma SY5Y neuron differentiation system [40] (Figure S1), therefore, the specificity for the role DAB2IP preferentially to the neuronal lineage from hMSCs. Flow cytometric analysis from 5-days subcultured cells showed that ~35% 3A6-hMSCs and 38% primary hMSCs of sh59 cells were positive for MAP2, which was significantly higher than shLuc control cells (Figure 3C).

To further investigate the effect of DAB2IP-KD on neuronal differentiation *in vivo*, we implanted primary hMSCs with or without knockdown of DAB2IP to the brain of SD rats for 3 weeks and examined MAP2 expression in tissue sections. Three-dimensional images from the colocalization study showed exogenous implanted hMSCs and MAP2-positive cells (red fluorescence) in implanted hMSCs (blue fluorescence) as indicated by the arrows (Figure 3E, left panel). The number of the implanted MAP2-positive cells in DAB2IP-KD hMSCs was significantly greater than in the mock-treated hMSCs (Figure 3E, right panel), suggesting that knockdown of DAB2IP in hMSCs might be a potential strategy to promote neuronal differentiation *in vivo*. In addition, to further confirm that DAB2IP-KD hMSCs had the potential to differentiate into functional neuronal cells, neuron-like function was validated by measuring the change in [Ca^2+^]i after stimulation with high K^+^. As shown in Figure 3F, elevated [Ca^2+^]i stimulated by high K^+^ was observed significantly in differentiated primary hMSCs and DAB2IP-KD cells. These results demonstrated that the DAB2IP-KD hMSCs could be promoted to differentiate into a functional neuronal lineage.

### Downregulation of DAB2IP correlates with growth inhibition of hMSCs

**Figure 4:**
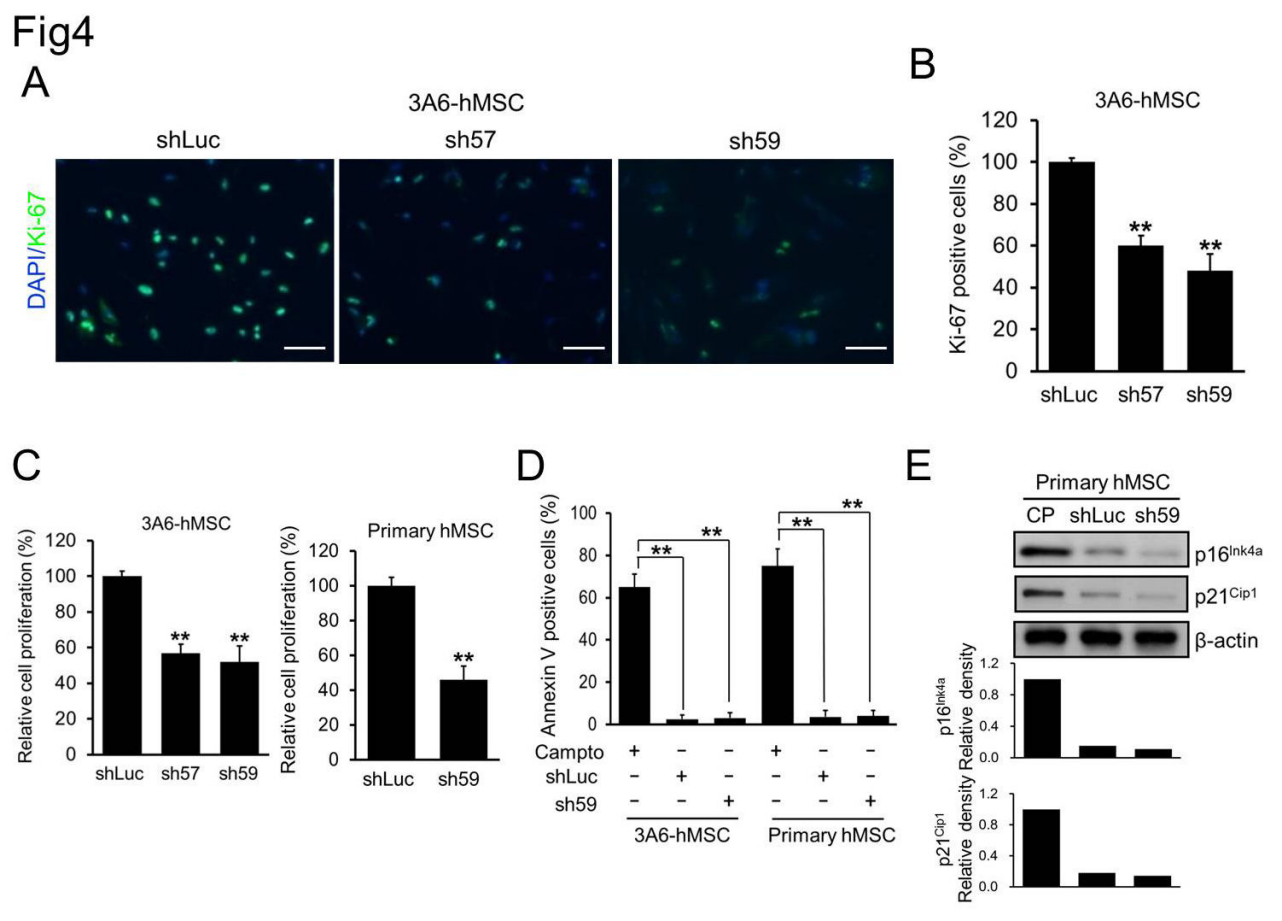
Correlation between the reduction of DAB2IP expression and hMSC proliferation. A. Immunofluorescence staining of Ki-67 (green) in shRNA-transfected 3A6-hMSCs. B. Quantification of Ki-67-positive shRNA-transfected 3A6-hMSCs. Bars represent mean ± SEM (**p < 0.01, *t* test). C. WST-1 cell survival and proliferation assay of shLuc, sh57 and sh59 cells. Bars represent mean ± SEM (**p < 0.01, *t* test). D. Percentage of apoptotic cells from each hMSC cell line treated with or without 6 µM camptothecin (Campto; positive control) for 4 h as determined by flow cytometry of annexin V-positive cells. Bars represent mean ± SEM (**p < 0.01, *t* test). E. Western blotting of factors involved in cellular senescence of hMSCs. Continuously passaged (CP) of hMSCs served as the positive control. The bar graphs (bottom) represent the relative density of p16^Ink4a^ and p21^Cip1^ as determined by scanning densitometric tracings.

During the subculture period, we noticed that the number of DAB2IP-KD cells gradually decreased in comparison with that of shLuc control cells. This was most likely due to the fact that DAB2IP downregulation not only promotes neuronal differentiation but also may inhibit the proliferation of 3A6-hMSCs. To test this possibility, we performed immunofluorescence staining of Ki67, a hallmark of cell proliferation, in these cells. The results indicated fewer Ki67-positive cells among sh57 and sh59 cells than in shLuc control cells on the day 5 of subculture (Figure 4A and B), which is consistent with the quantification of the percentages of Ki-67-positive cells among shLuc (100%), sh57 (60%) and sh59 (48%) (Figure 4B).

Moreover, the WST-1 cell survival and proliferation assay showed a similar inhibition of proliferation in DAB2IP-KD 3A6-hMSCs. Proliferation of sh57 and sh59 cells decreased by 58% and 52%, respectively, compared with shLuc control cells (Figure 4C), but there were no apparent differences with respect to apoptosis induction and cellular senescence, which activates the p16^Ink4a^ and p21^Cip1^ signaling pathways that function in the senescence of a variety of cells and in aging tissues (Figure 4D and E) [41]. Taken together, these results indicated that DAB2IP is a critical regulator of both neuronal differentiation and hMSCs proliferation.

### MtNeST, the first step for neuronal induction, of hMSCs is blocked by inhibition of GSK3β activity

There are at least two steps necessary for the neuronal induction of hMSCs to yield mature neurons: first, MtNeST must occur; second, cell cycle exit and differentiation. The induction of neuronal differentiation of hMSCs by KD of endogenous DAB2IP expression indicated that DAB2IP has the ability to promote the first step, namely MtNeST. We previously demonstrated that DAB2IP plays a role in the epithelial-to-mesenchymal transition (EMT) by modulating GSK3β activity [9]. We were therefore interested in dissecting whether the GSK3β signaling pathway is involved in MtNeST as well.

**Figure 5:**
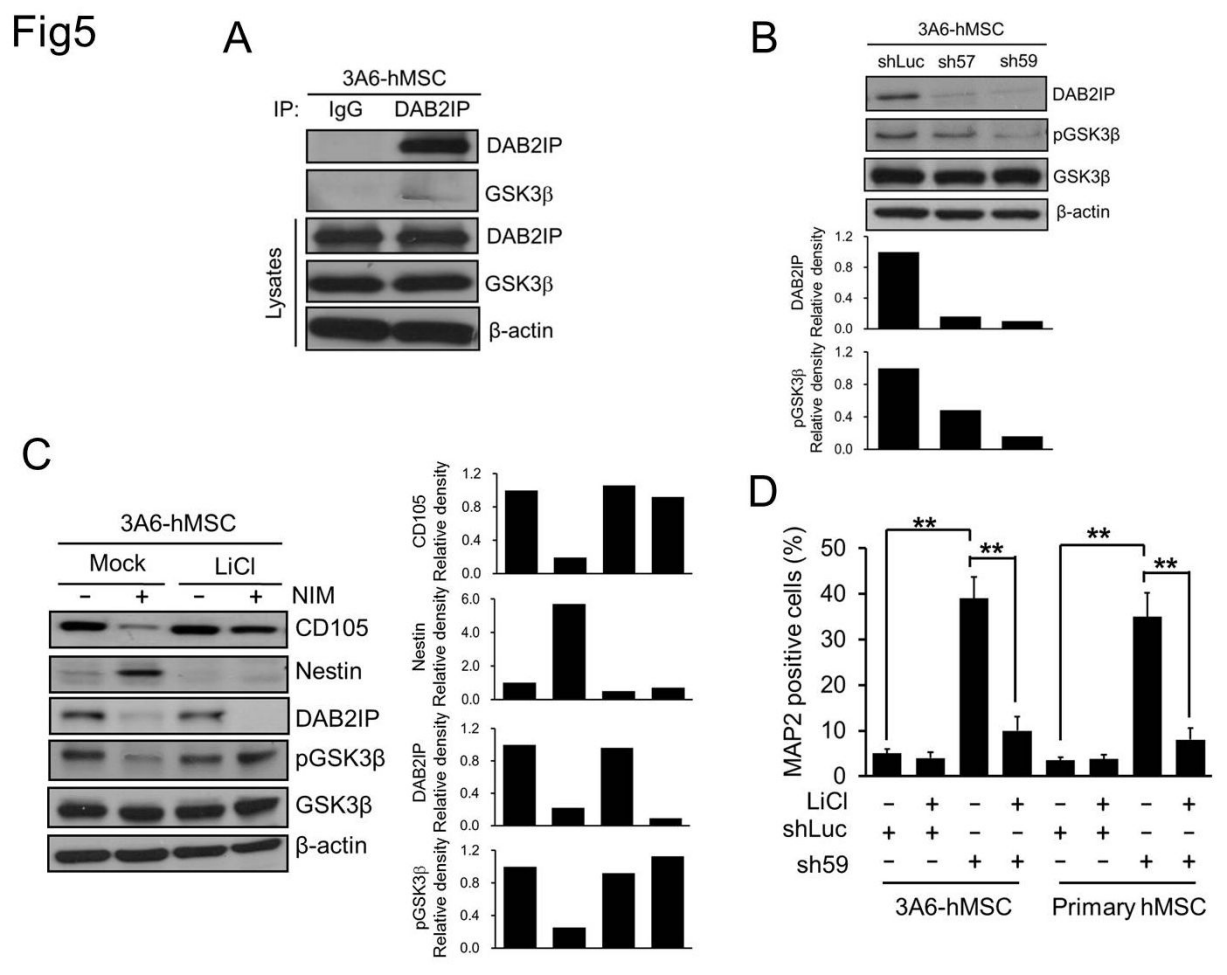
Effect of GSK3β on mesenchymal-to-neuroepithelial transition of hMSCs. A. Western blot analysis of whole-cell lysates from naïve 3A6-hMSCs. IP: immunoprecipitation. B. Western blot analysis of DAB2IP and Ser9 phosphorylated GSK3β (pGSK3β) in sh57 and sh59 DAB2IP-KD 3A6-hMSCs compared with shLuc control cells. The bar graphs (bottom) represent the relative density of DAB2IP, and pGSK3β as determined by scanning densitometric tracings. C. Western blot analysis for the MSC marker CD105 and the neuronal progenitor cell marker Nestin in naïve or LiCl-pretreated 3A6-hMSCs cultured with or without NIM. β-actin served as an internal control. The bar graphs (bottom) represent the relative density of CD105, Nestin, DAB2IP, and pGSK3β as determined by scanning densitometric tracings. D. Flow cytometry of MAP2-positive DAB2IP-KD hMSCs. Bars represent mean ± SEM (**p < 0.01, *t* test).

GSK3β protein expression was detected in the lysates of naïve 3A6-hMSCs, and GSK3β also co-immunoprecipitated with DAB2IP (Figure 5A), indicating that DAB2IP and GSK3β form a complex in 3A6-hMSCs. Furthermore, both sh57 and sh59 cells exhibited a reduction of Ser9-phosphorylated GSK3β (Figure 5B), implying elevated GSK3β activity in these cells [42]. This suggested that DAB2IP negatively regulates GSK3β activity in 3A6-hMSCs.

DAB2IP expression dramatically decreased at our earliest time point (i.e., first day) of neuronal induction, i.e., when MtNeST mainly occurred as evidenced by CD105 reduction and Nestin induction (neuroepithelial stem cell marker; Figures 1B and 5C, left panel); we therefore presumed that GSK3β activity would increase during this period. Indeed, pGSK3β levels decreased significantly after 24 h of culture in NIM (Figure 5C, left panel).

To elucidate whether an increase in GSK3β activity is necessary for MtNeST during the neuronal induction of 3A6-hMSCs, we pretreated 3A6-hMSCs with LiCl, a GSK3β inhibitor [42,43], for 16 h to inhibit GSK3β signaling. Then, cells were cultured with or without NIM for 24 h. Our results showed that pretreatment with LiCl significantly reduced the GSK3β activity in NIM for 24h cultured 3A6-hMSCs based on the elevation of pGSK3β (Figure 5C, right panel). Interestingly, under this condition, CD105 expression levels remained high, and no Nestin protein was induced by NIM despite the reduction in DAP2IP expression (Figure 5C). In addition, LiCl also significantly reduced the number of MAP2-positive cells, which were increased by DAB2IP-KD (Figure 5D).

Taken together, we concluded that the effect of DAB2IP on MtNeST during neuronal differentiation of hMSCs is mediated through the GSK3β signaling pathway.

## Discussion

Culturing hMSCs with NIM causes them to undergo sequential reprogramming steps, including MtNeST, cell cycle arrest, and neuronal differentiation, to become mature neurons. MtNeST is a very early event of neuronal induction. Many of the mesenchymal stem cells are believed to transform into neuroepithelial stem cells, as evidenced by the decreased expression of the MSC marker, CD105, and induction of the neuroepithelial stem cell marker, Nestin upon culture in NIM. DAB2IP expression decreases concurrently. Interestingly, MtNeST can be further promoted by DAB2IP KD using a lentivirus-mediated shRNA approach. Thus, our results demonstrate that decreased DAB2IP expression plays a crucial role in regulating MtNeST in hMSCs.

In 3A6-hMSCs, DAB2IP negatively regulates GSK3β activity, as evidenced by the reduction in Ser9-phosphorylated GSK3β in DAB2IP-KD 3A6-hMSCs. Suppression of GSK3β activity upon LiCl pretreatment abolished MtNeST induced by NIM in 3A6-hMSCs, suggesting that GSK3β activation is required for NIM-elicited MtNeST.

GSK3β is a serine/threonine kinase that mediates various signaling pathways, particularly the growth factor and Wnt signaling pathways [44]. GSK3β plays a key role in regulating neurogenesis, neuronal migration, polarization, and axon growth/guidance [45]. Interestingly, in metastatic prostate cancer cells DAB2IP appears to directly associate with GSK3β and form a complex with phosphatase 2A and axin, a component of the β-catenin destruction complex [9]. DAB2IP antagonizes Wnt-mediated EMT through the activation of GSK3β by decreasing Ser9 phosphorylation. Therefore, loss of DAB2IP expression facilitates EMT, leading to prostate cancer metastasis [9]. Of particular interest is that DAB2IP differentially regulates GSK3β activity in hMSCs and prostate cancer cells; DAB2IP activates GSK3β in prostate cancer cells but represses GSK3β in hMSCs. Despite the opposing effects of DAB2IP on the regulation of GSK3β activity in these two different cell types, a common effect of DAB2IP on modulating cell type transition (i.e., DAB2IP downregulation promoted cell fate transition) is observed in both models. Furthermore, it appears that GSK3β activation is involved in epithelial/neuroepithelial cell transition whereas GSK3β inactivation is involved in EMT. Taken together, we conclude that DAB2IP-GSK3β interaction is critical for cell fate transition between mesenchymal and epithelial/neuroepithelial stem cells.

During cortical neurogenesis, cortical neurons left from germinal zones (ventricular and subventricular zones) after their final mitosis migrate along radial glial fibers to form the cortical plate. On embryonic day 14.5 (E14.5) in the mouse cerebral cortex, *Dab2ip* mRNA is expressed either in the ventricular zone, where proliferative neuroprogenitor cells reside, or in the cortical plate, where differentiated mature neurons are positioned [10]. Our unpublished observations of Dab2ip protein expression in E11.5-E12.5 mouse brains have confirmed this biphasic expression pattern of DAB2IP in the early developmental stages of the cerebral cortex. In our present study, we showed that DAB2IP is critical for cell proliferation and neuronal differentiation in hMSCs. These findings suggest that loss of DAB2IP expression in cortical neuroprogenitor cells may play a crucial role in promoting cell cycle arrest and neuronal differentiation, as observed in hMSCs, in early developmental stages. Further studies are needed to address this possible role of Dab2ip in the early stages of cortical neurogenesis.

In the developing cortex and cerebellum, mouse Dab2ip regulates neuronal migration and neurite outgrowth [18,19]. Certain DAB2IP functions are closely related to its interacting protein, Dab1, the downstream adaptor protein of Reelin signaling [11,12]. It is well known that Reelin-Dab1 signaling plays an important role in neuronal migration and neuron positioning in the developing brain, particularly in the cerebral cortex, hippocampus, and cerebellum [8]. The laminar structure of the cerebral cortex is established by the inside-out sequence migration of cortical neurons generated at different developmental times. In Reelin- or Dab1-deficient mutant mice, the cortical neurons abnormally migrate by an opposite outside-in sequence [8,15]. In an attempt to manipulate endogenous Dab2ip expression levels in the mouse embryonic cortex, Lee et al. recently used an *in utero* electroporation method to introduce a Dab2ip expression plasmid or shRNA constructs. Their results showed that Dab2ip is required for cortical neuron migration, particularly for late-born neurons [18]. Dab2ip KD in E14.5 cortical progenitor cells restricts transfected neurons to the subventricular and intermediate zones without radial migration into the cortical plate at E17.5-E18.5. Furthermore, Dab2ip KD specifically reduced MAP expression and disrupted neurite development in primary cultured cortical neurons. The expression level of another neuronal cytoskeleton marker, Tuj-1, was not affected in Dab2ip-KD cortical neurons. These results different from our results for DAB2IP-KD 3A6-hMSCs; both Tuj-1 and MAP2 expressions was elevated as DAB2IP expression decreased. Taken together, the functions of DAB2IP in regulating the expression of neuronal cytoskeletonal proteins are likely cell type. The underlying molecular mechanisms require further study.

We presume that the expression level of DAB2IP during neurogenesis should be as dynamic as its endogenous biphasic expression pattern. Once DAP2IP levels decrease during cell cycle exit and neuronal differentiation, its expression should gradually increase and peak during determination of neuronal placement. In our hMSC neuronal induction system, DAB2IP KD triggered cell differentiation into Tuj-1-positive(early neuron marker) and MAP2-positive (late neuron marker) neuronal-like cells, but the percentage of MAP2-positive cells was significantly lower than that of Tuj-1-positive cells upon treatment with sh59. This may indicate that the reduction of DAB2IP is not sufficient or efficient for proper induction of neuronal differentiation. Alternatively, persistent DAB2IP KD may adversely affect proper neuronal differentiation, i.e., moderate DAB2IP expression is required for proper neuronal differentiation of hMSCs. However, our data, in accordance with other studies, demonstrate that DAB2IP is involved in regulating neuronal cytoskeletal protein expression during neurogenesis and neuronal induction [18,19].

In conclusion, our study shows that DAB2IP plays multiple roles in the neuronal induction of hMSCs, which implies a potential function for DAB2IP in the first step, i.e., MtNeST, of neurogenesis of the developing brain, especially the cerebral cortex.

## Supporting Information

Figure S1 **The expression of DAB2IP during differentiation.** A. expression of DAB2IP, osteogenic marker osteopontin (OPN) in hMSCs osteoblast (Ost) differentiation. B. expression of DAB2IP, neuron marker NSE in neuroblastoma SY5Y neuron differentiation.
(DOC)
