# Supplementary material for: Downregulation of DAB2IP Promotes Mesenchymal-To-Neuroepithelial Transition and Neuronal Differentiation of Human Mesenchymal Stem Cells
Source: PLoS One. 2013 Sep 20;8(9):e75884. doi: 10.1371/journal.pone.0075884 (PMC3779184; doi:10.1371/journal.pone.0075884)
Supplement: Figure S1 — The expression of DAB2IP during differentiation. A. expression of DAB2IP, osteogenic marker osteopontin (OPN) in hMSCs osteoblast (Ost) differentiation. B. expression of DAB2IP, neuron marker NSE in neuroblastoma SY5Y neuron differentiation. (DOC) [file pone.0075884.s001.doc]

**Supporting Information**


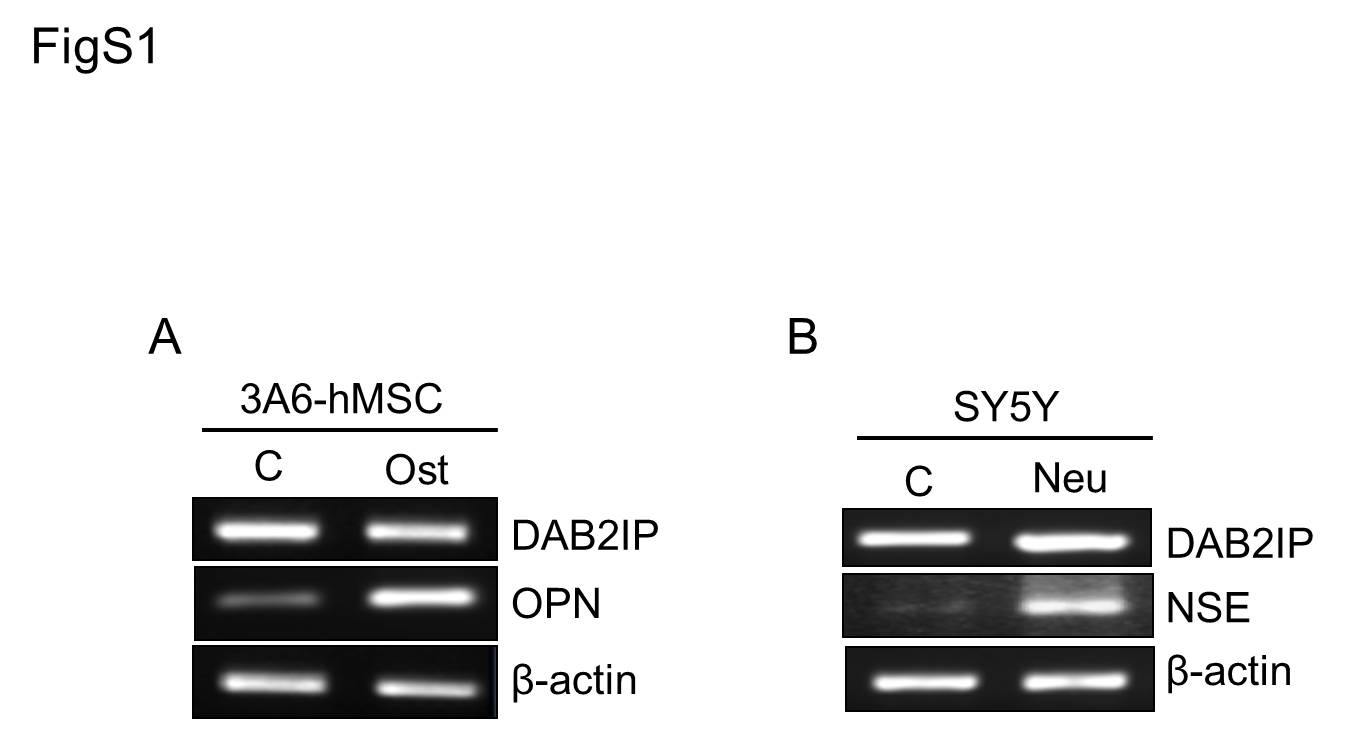
**Figure S1. The expression of DAB2IP during differentiation.** A. expression of DAB2IP, osteogenic marker osteopontin (OPN) in hMSCs osteoblast (Ost) differentiation. B. expression of DAB2IP, neuron marker NSE in neuroblastoma SY5Y neuron differentiation.
